# Mycoprotein: The Future of Nutritious Nonmeat Protein, a Symposium Review

**DOI:** 10.1093/cdn/nzz021

**Published:** 2019-04-04

**Authors:** Tim J A Finnigan, Benjamin T Wall, Peter J Wilde, Francis B Stephens, Steve L Taylor, Marjorie R Freedman

**Affiliations:** 1 Marlow Foods, Stokesley, UK; 2 College of Life and Environmental Science, University of Exeter, Exeter, UK; 3 Quadram Institute Bioscience, Norwich Research Park, Norwich, UK; 4 University of Nebraska-Lincoln, Food Allergy Research and Resource Program, Department of Food Science and Technology, Lincoln, NE; 5 Department of Nutrition, Food Science and Packaging, San Jose State University, San Jose, CA

**Keywords:** mycoprotein, Quorn, nutrition and health, alternate protein, protein synthesis, sustainable protein

## Abstract

Mycoprotein is an alternative, nutritious protein source with a meat-like texture made from *Fusarium venenatum*, a naturally occurring fungus. Its unique method of production yields a significantly reduced carbon and water footprint relative to beef and chicken. Mycoprotein, sold as Quorn, is consumed in 17 countries, including the United States. In line with current dietary guidelines, mycoprotein is high in protein and fiber, and low in fat, cholesterol, sodium, and sugar. Mycoprotein may help maintain healthy blood cholesterol levels, promote muscle synthesis, control glucose and insulin levels, and increase satiety. It is possible that some susceptible consumers will become sensitized, and subsequently develop a specific allergy. However, a systematic evidence review indicates that incidence of allergic reactions remains exceptionally low. Mycoprotein's nutritional, health, and environmental benefits affirms its role in a healthful diet. Future research that focuses on the long-term clinical benefits of consuming a diet containing mycoprotein is warranted.

The objectives of this symposium were to describe the source, processing, and environmental impact of mycoprotein; describe mycoprotein's key nutritional attributes for human health; and examine areas of emerging mycoprotein research related to worldwide health issues.

Dr Tim Finnigan, Chief Scientific Advisor for Marlow Foods (Stokesley, UK) described mycoprotein's discovery, processing, approval as a food ingredient, introduction into the food supply, and its environmental footprint. He explained that >50 years ago, the “green revolution” inspired British scientists, led by Lord Rank, to find a new, sustainable protein source that could convert plentiful starch into less plentiful protein. He described how scientists collected and tested >3000 soil organisms from around the world until they discovered that *Fusarium venenatum*, a filamentous microfungus found originally in a field in Buckinghamshire, UK, made this conversion (1). It took 20 years of research and development to produce the mycoprotein, via the continuous fermentation of *F. venenatum* followed by steaming, chilling, and freezing of the RNA-reduced biomass (2). This process results in a high-protein and high-fiber food with a high degree of fibrosity through fiber assembly. When examined under a microscope mycoprotein that has been prepared in this way has a texture similar to that of chicken breast (3). By dry weight, mycoprotein is typically 45% protein and 25% fiber (1). **Table 1** provides mycoprotein's nutrient content in its food ingredient form.

**TABLE 1:** Nutritional Composition of Mycoprotein per 100 g (wet weight)^1^

Nutrient	Quantity
Energy, kcal	85
Protein, g	11
Total fat, g	2.9
Saturated fatty acids, g	0.7
Monounsaturated fatty acids, g	0.5
Polyunsaturated fatty acids, g	1.8
Total carbohydrate, g	3.0
Sugars, g	0.5
Dietary fiber, g	6.0
Vitamin B-12, μg	0
Sodium, mg	5.0
Cholesterol, g	0
Iron, mg	0.5
Zinc, mg	9.0
Selenium, μg	20

1 Source: https://www.mycoprotein.org/health-nutrition/nutritional-composition.

Eventually, mycoprotein was produced in sufficient quantities to be tested for use as a commercial food ingredient. In 1983, after a 10-y evaluation, the UK Ministry of Agriculture, Fisheries and Food approved mycoprotein for food use. Two years later, a savory pie became the first mycoprotein-containing retail product sold in the UK under the brand name Quorn (Marlow Foods). In 2002, the US FDA designated mycoprotein as “Generally Recognized as Safe” and 7 Quorn products were introduced into the US food supply (4). Currently, Quorn is sold in 17 countries as an ingredient in frozen or refrigerated food products with an estimated 5 billion servings consumed worldwide since launch. Since a small amount of egg albumin is

added as a binder at the end of processing, Quorn foods cannot be classed as vegan. However, recent breakthroughs in food technology are now allowing an increasing proportion of the Quorn portfolio to be sold without egg and these have received approval from the Vegan Society. This trend is set to continue. In addition, recent investigations into technologies such as high-pressure homogenization now offer the possibility that mycoprotein can be used to create new vegan formats, flavors, and uses, notably applications in the growing nondairy milk and desserts category.

Production of mycoprotein through fermentation provides a distinct and relatively benign environmental footprint. These environmental impacts have been quantified through the use of techniques such as lifecycle analysis that conform to international standards and whose conclusions are independently audited and certified by the Carbon Trust (5). Comparison of Quorn grounds with beef, for example, show ≥10 times less embedded carbon, land, and water use (6). In addition, because mycoprotein is grown with the carbohydrate fraction of crops as the fermentation substrate, the process can be considered to give rise to an overall net gain in protein. This is because the original protein from the crops is not used in the fermentation of mycoprotein and is thus conserved. This is unlike many other food protein production systems, most notably animal protein, which result in a net decrease in protein because of their inherent inefficiencies. Thus, as we look at the increasing difficulties of assuring a global sustainable food future, mycoprotein fermentation technology offers an important new tool with which to meet this challenge.

Dr Benjamin Wall, Senior Lecturer in Nutritional Physiology, Department of Sport and Health Sciences, University of Exeter, UK, explained the potential role of mycoprotein in supporting skeletal muscle maintenance and reconditioning. Although skeletal muscle can be increased through conditioning, he noted that the worldwide aging population is facing sarcopenia, the age-related loss of muscle mass. By 80 y of age, body composition changes result in an almost 50% loss of muscle mass (from 48% to 25%) and an 84% increase in body fat (from 19% to 35%) (7).

Dr Wall described how stable isotopes are used to measure in vivo rates of muscle protein synthesis. Using this method, nutrition coupled with exercise was demonstrated to have a synergistic anabolic effect on muscle protein synthesis rates in healthy young men, facilitating reconditioning (i.e., adaptation) (8). In line with this, protein intake above the RDA was demonstrated to augment the adaptation to training (9,10).

With aging, however, muscle protein metabolism changes. Anabolic resistance to dietary protein in older adults appears to be a key factor underlying sarcopenia (11). Additionally, older adults require a larger amount of protein per meal to optimize muscle protein synthesis rates (12). Specifically, to offset sarcopenia, adults aged 66–87 and >87 y may require a daily protein intake of 1.2 and 1.6 g/kg body weight (13,14), respectively, which is a 50% and 100% increase compared with the recommended intake of 0.8 g/kg body weight for younger adults. Considering the global demands for protein in a rapidly increasing aging population, it is important to determine sustainable ways to meet future protein needs.

Assessment of in vivo muscle protein synthesis rates after consumption of meat, eggs, milk (casein and whey), free amino acids, soy, and other plant-based protein sources indicates that the postprandial anabolic potential of a particular dietary protein is primarily a function of its amino acid composition. Other factors include the postprandial insulin response, digestion and absorption kinetics, and amino acid bioavailability (15,16).

Mycoprotein is rich in essential amino acids (EAAs). Its EAA composition as a percentage of total protein is 41%, similar to spirulina. This value is higher than most other commonly consumed plant-based proteins. Although almost all animal sources of protein have a higher percentage of EAA relative to total protein (≤52% for whey), mycoprotein's composition compares favorably with human muscle (at 45%) (17). As a sustainable, plant-based protein, mycoprotein may play an important role in meeting the protein needs of an aging population.

To assess the effect of mycoprotein ingestion on plasma EAA and BCAA concentrations, a randomized, single-blind, crossover, dose-response study in 12 healthy young males was conducted (18). Postprandial plasma EAA and BCAA concentrations were assessed in the fasting state, and at regular intervals for 4 h after subjects consumed either 20 g milk protein, a “mass-matched” bolus of mycoprotein, a protein-matched bolus of mycoprotein, or 60 or 80 g boluses of mycoprotein. Results indicate a significant effect of time, and time × treatment. Mycoprotein ingestion resulted in slower but more sustained plasma EAA and BCAA concentrations compared with milk when protein matched. Increasing the dose of mycoprotein amplified these effects, with some evidence of a plateau at 60–80 g (18).

Dr Wall noted that ongoing research supports the synergistic effect of mycoprotein plus exercise on muscle protein synthesis. His future research will focus on mycoprotein's ability to stimulate muscle protein synthesis rates in older adults; differences in muscle protein synthesis responses between mycoprotein and animal-derived protein sources in rested and exercised muscle in healthy young athletes; and mycoprotein as a component of diets that support training adaptations in young athletes or muscle reconditioning in active older adults.

Dr Peter Wilde, Professor in the Food Innovation & Health Programme, Quadram Institute Bioscience, Norwich, UK, provided insights into the role of the food matrix structure on digestibility and bioavailability. Structure is a critical component of food. It not only affects food quality (e.g., taste, texture and shelf life), but it also affects digestion and absorption of nutrients that may have health benefits (19,20). Controlling or delaying digestion by consuming a food with resistant starch, for example, can result in a reduced glycemic response, reduced appetite, and change in colonic microbiota (21). These changes may decrease the risk for type 2 diabetes.

Dietary fiber, found in cell walls, remains undigested in the small intestine. Fiber can encapsulate and control the release and absorption of nutrients in plant-based foods (20,22). The dietary fiber in mycoprotein is naturally occurring and is comprised of approximately one-third chitin (*N*-acetylglucosamine) and two-thirds β-glucan (1,3-glucan and 1,6-glucan) (23).

Ongoing research aims to determine potential mechanisms underpinning nutrient bioavailability from mycoprotein by examining cell wall structural changes during processing and digestion. To study the effect of structure, the mycoprotein particle size was decreased from 140 to <20 μm through incubation, homogenization, sonication, or grinding with glass beads. Smaller particles released the largest percentage of protein (30%). However, to reduce the particle size, intensive treatments such as sonication and grinding were required to disrupt cells and release protein, indicating that mycoprotein's cell walls are effective at encapsulating proteins and are resistant to disruption. In vitro digestion studies further reveal a total protein release of 51% from the mycoprotein structure, primarily occurring in the small intestine. However, fluorescence microscopy indicates that even after postintestinal digestion, cell walls appear to be intact and unchanged. Therefore, a simple in vitro digestion results in a greater release of protein than intensive physical treatments such as ultrasound. This suggests that the high protein bioavailability observed by Dr Wall's group is due to the action of the digestive conditions on the mycoprotein cell wall properties. It is thus possible that digestion instigates a significant change in the porosity of mycoprotein's cell walls, enabling either protein release or access by the digestive enzymes. Further research is needed to determine the fundamental nature of these changes, and how and why they lead to the high protein bioavailability.

Dr Francis Stephens, Associate Professor of Nutritional Physiology, University of Exeter, UK, discussed the positive effects of mycoprotein consumption on glycemia, insulinemia, lipidemia, and short-term energy intake. He described one of the early studies that reported lean subjects who consumed a 250-mL milkshake containing 17 g mycoprotein had a 13% reduction in glycemia 1 h postingestion (24). He then discussed a later randomized trial that was conducted in overweight and obese subjects who consumed low- (44 g), medium- (88 g) or high- (132 g) protein meals containing isocaloric amounts of mycoprotein or chicken (24). The carbohydrate and fat content of the meals were similar, but fiber was higher in all mycoprotein compared to matched chicken meals. Results indicated that mycoprotein meals had no effect on glycemic response, but they elicited a reduced insulin response at every level compared with chicken. Energy intake at an ad libitum lunch following test meals was lower only at the highest level of mycoprotein (25).

Stephens noted that ongoing research by Coelho and colleagues at the University of Exeter is examining the metabolic effects of a 1-wk high-mycoprotein diet. Pilot study results suggest no effect of mycoprotein on blood glucose or serum insulin concentrations during an oral glucose tolerance test. The NMR metabolomics biomarker approach revealed only a nonsignificant (*P* = 0.06) decrease in the plasma glucose response, whereas a significant robust decrease in plasma cholesterol, predominantly in the smaller lipoprotein particles, was seen. These results support earlier findings that showed cholesterol reductions following mycoprotein consumption over 3- and 8-wk periods (26,27).

The cholesterol-lowering effects of mycoprotein appear to be due to its high fiber content, coupled with its unique composition. Chitin and β-glucans create a fibrous, 88% insoluble matrix that may be a factor in delaying BCAA or glucose absorption, and impairing cholesterol or bile absorption. Further, bacterial metabolism of fiber in the gut may result in a greater production of SCFAs that may affect cholesterol synthesis or lipolysis.

Mycoprotein appears an appropriate dietary component for metabolic health. Studies that use more robust measures such as the euglycemic hyperinsulinemic clamp are needed to confirm results regarding improved insulin sensitivity. Continued use of metabolomics biomarker approaches and long-term feeding studies are warranted.

Dr Steve Taylor, Food Allergy Research and Resource Program, University of Nebraska–Lincoln, NE, discussed the initial approval of mycoprotein as a food ingredient in the United States as well as issues relating to its safety in the food supply. He explained that Miller and Dwyer (28) reviewed the basis for “Generally Recognized as Safe” approval in the United States, and that following a review of all available information in 1998, the Expert Panel concluded that, to a reasonable scientific certainty, “Mycoprotein is a safe and suitable ingredient for use in food as a source of protein in the diet” (28).

Taylor noted that the main issue relating to food safety relates to allergenicity, and reminded the audience that ingestion of any novel dietary protein, including mycoprotein, may elicit an allergic reaction. He explained how susceptible consumers will become sensitized and develop mycoprotein-specific IgE antibodies. Since US approval in 2002, 5 individual case reports of mycoprotein-specific allergy (29–33) and 1 case of food protein–induced enterocolitis syndrome—a type of food allergy affecting the gastrointestinal (GI) tract—have appeared in the clinical literature (34). The best-investigated case reported that a 41-y-old male with an allergic reaction to mycoprotein had a positive skin test and serum IgE to *Fusarium* sp. (30)

In 2015, the York Health Economics Consortium conducted a systematic review of allergic reactions to mycoprotein. Among 30 experimental studies, investigators confirmed only 2 reactions. The Consortium concluded: “An assessment of the published and unpublished evidence indicates that reported intolerance reactions to Quorn are very low relative to common allergenic foodstuffs. Adverse reactions of any kind to Quorn are rare and for the vast majority of individuals, Quorn represents a safe foodstuff. … Rare cases of true allergy to Quorn do occur, as for many other common foods” (35).

Taylor explained how Marlow Foods has tracked worldwide consumer complaints since mycoprotein entered the UK marketplace in 1985. Examination of all reported adverse reaction complaints, without an attempt to differentiate between genuine and coincidental, indicates that the incidence of worldwide adverse reactions reported to the company over >30 y remains exceptionally low. Taylor conducted an in-depth analysis of the Marlow Foods database of reported illnesses (RIs) over the most recent 15-y period (2003–2017). RIs were characterized as either GI intolerance or possible IgE-mediated, true allergy, with symptoms beyond GI. The possible frequency of both categories of RI was determined as a function of yearly packages of Quorn sold and of servings potentially consumed. Because the analysis was based on self-reported data, and the assumption was that any RI was attributed to mycoprotein, results likely overestimate the true frequency and should be considered a worst-case scenario.

Over the 15-y period, the frequency of RIs per packages sold was 1 RI per 683,665 packages or 1 RI per 1.85 million servings. The frequency of possible IgE-mediated reactions (true allergy) was 1 per 8.99 million packages or 1 per 24.3 million servings. Over this 15-y period, the database recorded a total of 2327 RIs, of which 177 are reports where symptoms are “allergic” in their description. Forty-two of the total RI reports suggest some form of medical treatment was sought, a frequency of 1 in 37.9 million packages sold. Examination of these self-reported data, both real and extrapolated, indicates that although mycoprotein may be allergenic, the rate of allergic reactions is extremely low—1 in 9 million packages.

Novel sources of dietary protein will always provoke allergic reactions in some consumers. With the surge of novel protein sources (e.g., pea, lupin, insects, and hemp) entering the US marketplace, and the increased intake of existing protein sources (e.g., casein, whey, and soy), it is likely that rates of allergic reactions to novel protein sources will increase. Although the rate of such reactions compared with those elicited by mycoprotein is unknown, it is well known that allergies to milk, soy, legumes, and crustacean shellfish (which are related to insects) are common.

The Marlow Foods database indicates that 92% of RIs are only associated with GI symptoms. An Expert Panel comprised of scientists from the United States, the United Kingdom, and Australia was convened in 2011 to review adverse reactions and possible causal mechanisms. The Panel concluded that GI symptoms were likely due to mycoprotein's high fiber content. They hypothesized that in certain individuals under certain conditions, consuming mycoprotein could speed up the normal transit time of foods from the small intestine to the large intestine. This could, in turn, cause rapid fermentation of mycoprotein fiber in the large intestine, causing GI distress symptoms. Predisposing factors include usual dietary fiber intake, imbalance of GI bacteria, or ≥1 manifestations of irritable bowel syndrome. The Panel concluded that mycoprotein is safe and suitable for consumers, including the elderly and those who might benefit from its fiber and protein content (36).

The Center for Science in the Public Interest (CSPI) has collected anecdotal reports on adverse reactions (both GI complaints and allergic reactions) to mycoprotein through its website since 2002. Between 2002 and 2014, the number of incidents occurring in the United Kingdom that were reported to CSPI (1095) and Marlow Foods (11181) was comparable. During this time, there were twice as many incidents occurring in the United States reported to CSPI (683) as compared with Marlow Foods (356), but fewer worldwide incidents (229 compared with 308, CSPI compared with Marlow, respectively). CSPI claims that mycoprotein is unsafe, even though they fail to present the denominator that would allow for an estimate of the frequency of unsafe reactions. CSPI also indicates that 63% of respondents had adverse reactions on their first exposure to mycoprotein. Because individuals have to be sensitized first, it is more likely that these reactions were GI in nature, and unlikely to be allergic reactions. Additionally, self-reported data should be treated with caution.

Novel food sources of protein introduced into the US food supply (such as lupine, peas, canola protein isolate, insects, wheat protein isolate, and mycoprotein) can become allergenic. Predictors of allergenic potential include knowledge that the food source is allergenic when consumed in limited quantities (e.g., cottonseed protein) or that it is allergenic in another country where it is more commonly consumed (e.g., buckwheat among Japanese and Koreans). Other predictors include whether the food is genetically related to known allergenic foods (e.g., canola protein is very closely related to mustard) or if the food contains a potentially cross-reactive protein (e.g., insects and crustacean shellfish). Whether the protein is readily digestible is also a predictor. From an allergy perspective, mycoprotein may be among the safest novel protein sources on the market.

In conclusion, this symposium confirmed that mycoprotein is a nutritious, sustainable protein source in line with current dietary guidelines. Research suggests that mycoprotein may help maintain healthy blood cholesterol levels and promote muscle synthesis, control blood glucose and insulin, and increase satiety. As a protein, it is possible that some susceptible consumers will become sensitized, and subsequently develop a specific allergy. However, a systematic review of the evidence indicates that the incidence of allergic reactions to mycoprotein remains exceptionally low. In the opinion of the speakers, mycoprotein's nutritional, health, and environmental benefits affirm its safety and role in a healthful diet. Future research that focuses on long-term clinical benefits of consuming a diet containing mycoprotein is warranted.

## Notes

Supported by Marlow Foods (BTW);

UK Biotechnology and Biological Sciences Research Council (BBSRC), Irish Government, Danish Government, Marlow Foods, and Mondelez International (PJW); and

Ketchum Communications (MRF).

Author disclosures: TJAF is Chief Scientific Officer, Marlow Foods; BTW received travel funds and meals at the meeting from Marlow Foods; PJW is primary supervisor for a PhD studentship funded by Marlow Foods; received travel funds and meals at the meeting from Marlow Foods; FBS received travel funds and meals at the meeting from Marlow Foods; SLT received travel funds and an honorarium from Marlow Foods; MRF, no conflicts of interest.

Supplement disclosure statement: Presented as a preconference symposium at the American Society of Nutrition meeting held in Boston, MA, June 8, 2018. The conference was organized by Cathy Kapica, PhD of the Awegrin Institute with help from Wendy Weiss, Ketchum Communications, and supported by a grant from Marlow Foods. Publication costs for this supplement were defrayed in part by the payment of page charges by Marlow Foods. The opinions expressed in this publication are those of the authors and are not attributable to the sponsors or the publisher, Editor, or Editorial Board of *Current Developments in Nutrition*.

Abbreviations used: CSPI, Center for Science in the Public Interest; EAA, essential amino acid; GI, gastrointestinal; RI, reported illness; YHEC, York Health Economics Consortium.

## References

[1] Finnigan TJA Mycoprotein: origins, production and properties. In: Philips GO Williams PA, editors. *Handbook of Food Proteins* Cambridge (UK): Woodhead Publishing; 2011 p. 335–52.

[2] Mycoprotein.org. How is mycoprotein made? [Internet]. c2019 [cited January 5, 2019]. Available from: https://www.mycoprotein.org/how-is-mycoprotein-made.

[3] Mycoprotein.org. What is mycoprotein? The mycoprotein story [Internet]. c2019. [cited January 5, 2019] Available from https://www.mycoprotein.org/what-is-mycoprotein.

[4] Marlow Foods. GRAS Notification for Mycoprotein. (submitted as: US FDA, 2002—GRN 091).: Stokesley (UK): Marlow Foods Ltd.

[5] The Carbon Trust. Quorn-product carbon footprinting and labeling [Internet]. [cited January 5, 2019]. Available from: https://www.carbontrust.com/our-clients/q/quorn-product-carbon-footprinting-and-labeling/.

[6] Finnigan TJA, Needham L, Abbot C Mycoprotein: a healthy new protein with a low environmental impact. In: Nadathur SR Wanasundara JPD Scanlin L editors. Sustainable Protein Sources. London: Academic Press; 2017 p. 305–25.

[7] Short KR, Nair SK. The effect of age on protein metabolism. Curr Opin Clin Nutr Metab Care 2000;3:39–44. 10642082 10.1097/00075197-200001000-00007

[8] Wall BT, Burd NA, Franssen R, Gorissen SH, Snijderss T, Senden JM, Gijsen AP, van Loon LJ Presleep protein ingestion does not compromise the muscle protein synthetic response to protein ingested the following morning. Am J Physiol Endocrinol Metab 2016;311:E964–73. 27780822 10.1152/ajpendo.00325.2016

[9] Cermak N, Res P, de Groot L, Saris W, van Loon L Protein supplementation augments the adaptive response of skeletal muscle to resistance-type exercise training: a meta-analysis. Am J Clin Nutr 2012;96:1454–64. 23134885 10.3945/ajcn.112.037556

[10] Morton R, Murphy K, McKellar S, Schoenfeld BJ, Henselmans M, Helms E, Aragon AA, Devries MC, Banfield L, Krieger JW et al. A systematic review, meta-analysis and meta-regression of the effect of protein supplementation on resistance training-induced gains in muscle mass and strength in healthy adults. Br J Sports Med 2018;52:376–84. 28698222 10.1136/bjsports-2017-097608 PMC5867436

[11] Wall B, Gorissen S, Pennings B, Koopman R, Groen BB, Verdijk LB, van Loon LJ Aging is accompanied by a blunted muscle protein synthetic response to protein ingestion. PLoS One 2016;10:e0140903. 10.1371/journal.pone.0140903 PMC4633096 26536130

[12] Moore D, Churchward-Venne T, Witard O, Breen L, Burd NA, Tipton KD, Phillips SM Protein ingestion to stimulate myofibrillar protein synthesis requires greater relative protein intakes in healthy older versus younger men. J Gerontol A Biol Sci Med Sci 2015;70:57–62. 25056502 10.1093/gerona/glu103

[13] Meng X, Zhu K, Devine A, Kerr D, Binns C, Prince R A 5-year cohort study of the effects of high protein intake on lean mass and BMC in elderly postmenopausal women. J Bone Miner Res 2009;24:1827–34. 19419320 10.1359/jbmr.090513

[14] Houston D, Nicklas B, Ding J, Harris TB, Tylavsky FA, Newman AB, Lee JS, Sahyoun NR, Visser M, Kritchevsky SB et al. Dietary protein intake is associated with lean mass change in older, community-dwelling adults: The Health, Aging, and Body Composition (Health ABC) Study. Am J Clin Nutr 2008;87:150–5. 18175749 10.1093/ajcn/87.1.150

[15] Pennings B, Boirie Y, Senden J, Gijsen A, Kuipers H, van Loon L Whey protein stimulates postprandial muscle protein accretion more effectively than do casein and casein hydrolysate in older men. Am J Clin Nutr 2011;93:997–1005. 21367943 10.3945/ajcn.110.008102

[16] Burd N, Gorissen S, van Vliet S, Snijders T, van Loon L Differences in postprandial protein handling after beef compared with milk ingestion during postexercise recovery: a randomized controlled trial. Am J Clin Nutr 2015;102:828–36. 26354539 10.3945/ajcn.114.103184

[17] van Vleit S, Burd JA, van Loon LJC The skeletal muscle anabolic response to plant-versus animal-based protein consumption. J Nutr 2015;145:1981–91. 26224750 10.3945/jn.114.204305

[18] Dunlop MV, Kilroe SP, Bowtell JL, Finnigan TJA Mycoprotein represents a bioavailable and insulinotropic non-animal derived dietary protein source: a dose response study. Br J Nutr 2017;118:673–85. 29017627 10.1017/S0007114517002409

[19] Wahlqvist ML Food structure is critical for optimal health. Food Funct 2016;7(3):1245–50. 26667120 10.1039/c5fo01285f

[20] Capuano E, Pellegrini N An integrated look at the effect of structure on nutrient bioavailability in plant foods. J Sci Food Agric 2019;99(2):493–8. 30066376 10.1002/jsfa.9298

[21] Birt DF, Boylston T, Hendrich S, Jane JL, Hollis J, Li L, McClelland J, Moore S, Phillips GJ, Rowling M et al. Resistant starch: promise for improving human health. Adv Nutr 2013;4(6):587–601. 24228189 10.3945/an.113.004325 PMC3823506

[22] Grundy MML, Grassby T, Mandalari G, Waldron KW, Butterworth PJ, Berry SE, Ellis PR Effect of mastication on lipid bioaccessibility of almonds in a randomized human study and its implications for digestion kinetics, metabolizable energy, and postprandial lipemia. Am J Clin Nutr 2015;101(1):25–33. 25527747 10.3945/ajcn.114.088328 PMC4266890

[23] Denny AEA Mycoprotein and health. Nutr Bull 2008;33:298–310.

[24] Turnbull WH, Ward T Mycoprotein reduces glycemia and insulinemia when taken with an oral glucose tolerance test. Am J Clin Nutr 1995;61:135–40. 7825525 10.1093/ajcn/61.1.135

[25] Bottin JH, Swann JR, Cropp E, Chambers ES, Ford HE, Ghatei ME, Frost GS Mycoprotein reduces energy intake and postprandial insulin release without altering glucagon-like peptide-1 and peptide tyrosine-tyrosine concentrations in healthy overweight and obese adults: a randomised-controlled trial. Br J Nutr 2016;116:360–74. 27198187 10.1017/S0007114516001872 PMC4910676

[26] Turnbull WH, Leeds AR, Edwards GD Effect of mycoprotein on blood lipids. Am J Clin Nutr 1990;52(4):646–50. 2169701 10.1093/ajcn/52.4.646

[27] Turnbull WH, Leeds AR, Edwards GD Mycoprotein reduces blood lipids in free-living subjects. Am J Clin Nutr 1992;55:415–19. 1734679 10.1093/ajcn/55.2.415

[28] Miller SA, Dwyer JT Evaluating the safety and nutritional value of mycoprotein. Food Technol 2001;55:42–7.

[29] Kalona SJ, Kaminski ER Sensitivity to Quorn mycoprotein (*Fusarium venenatum*) in a mould allergic patient*.* J Clin Pathol 2002;55:876–7. 10.1136/jcp.55.11.876-a PMC1769805 12401831

[30] Hoff M, Trueb RM, Ballmer-Weber BK, Vieths S, Wuethrich B Immediate-type hypersensitivity reaction to ingestion of mycoprotein (Quorn) in a patient allergic to molds caused by acidic ribosomal protein P2. J Allergy Clin Immunol 2003;111(5):1106–10. 12743577 10.1067/mai.2003.1339

[31] Van Durme P, Ceuppens JL, Cadot P Allergy to ingested mycoprotein in a patient with mold spore inhalant allergy. J Allergy Clin Immunol 2003;112:452–4. 12897757 10.1067/mai.2003.1613

[32] Khurana S, Jenkins JS Exercise induced anaphylaxis to Quorn mycoprotein: C12. Clin Exp Allergy 2007;37:1875.

[33] Dzeladini L, Chan D, Kummerow M A case report of mycoprotein allergy. Int Med J 2017;47(Suppl 5):17.

[34] Tan JA, Smith WB. Non-IgE-mediated gastrointestinal food hypersensitivity syndrome in adults. J Allergy Clin Immunol Pract 2014;2:355–7. 24811033 10.1016/j.jaip.2014.02.002

[35] York Health Economics Consortium. Systematic Review of Allergic Reactions to Quorn and 14 Common Foods. Presented to Marlow Foods, 2015, unpublished.

[36] Expert Panel. *Mycoprotein: Report of the Expert Panel, Unpublished Report* Stokesley (UK): Marlow Foods; 1998, unpublished.

